# Role of Ultrasound Elastography and Contrast-Enhanced Ultrasound (CEUS) in Diagnosis and Management of Malignant Thyroid Nodules—An Update

**DOI:** 10.3390/diagnostics15050599

**Published:** 2025-03-01

**Authors:** Carolina Solomon, Diana-Raluca Petea-Balea, Sorin Marian Dudea, Ioana Bene, Cristina Alina Silaghi, Manuela Lavinia Lenghel

**Affiliations:** 1 Department of Radiology, “Iuliu Hatieganu” University of Medicine and Pharmacy, 400012 Cluj-Napoca, Romania; carolinasolomon12@gmail.com (C.S.); sdudea1@gmail.com (S.M.D.); ioanaboca90@yahoo.com (I.B.); lenghel.manuela@gmail.com (M.L.L.); 2 Department of Endocrinology, “Iuliu Hatieganu” University of Medicine and Pharmacy Cluj-Napoca, 8 Victor Babes Street, 400012 Cluj-Napoca, Romania; alinasilaghi@yahoo.com

**Keywords:** thyroid cancer, contrast-enhanced ultrasound, elastography, indeterminate thyroid nodules, thermal ablation procedures

## Abstract

The aim of this paper is to highlight the combined role of ultrasound elastography and contrast-enhanced ultrasound in terms of diagnosis, staging, and follow-up of the post-treatment response. Contrast-enhanced ultrasound (CEUS) and ultrasound elastography are natural extensions of conventional USs that have created new opportunities, facilitating the implementation of multiparametric ultrasounds in the characterization of thyroid nodules, in risk stratification, and in the selection of nodules that request Fine Needle Aspiration (FNA), management, and follow-up of the nodules with indeterminate cytology, evaluation of pre-operative prognostic features, and treatment efficiency.

## 1. Introduction

Thyroid nodule prevalence in the general population varies greatly by country and has been estimated to reach 50–67% depending on the detection method. Ultrasonography (US) is a non-invasive, highly accurate, and cost-effective diagnostic tool for thyroid lesion assessment. Most thyroid nodules are benign or lack ultrasound characteristics that would indicate malignancy, making them largely clinically insignificant even though high prevalence rates indicate a substantial burden of disease [1,2,3]. Unfortunately, B-mode US cannot always provide proper differentiation between benign and malignant thyroid nodules; although there are a number of sonographic features suggesting high-risk malignancy, such as microcalcifications, solid structure, hypoechoic appearance, irregular demarcation, and a ‘taller-than-wide’ shape, no single characteristic is sufficiently sensitive and specific to rule out or validate thyroid cancer [4]. For this reason, particularly in the last decade, it has become increasingly important to support new ultrasound techniques to optimize the interpretation of US images and minimize unnecessary invasive diagnostic procedures [5,6].

The contrast-enhanced ultrasound (CEUS) and US elastography are natural extensions of conventional USs that have created new opportunities, facilitating the implementation of multiparametric ultrasounds in the characterization of thyroid nodules as recommended by most experts and several societies. The role of multiparametric US has been extended to also assess risk stratification and the selection of nodules that request Fine Needle Aspiration (FNA), management, and follow-up of the nodules with indeterminate cytology, evaluation of pre-operative prognostic features, and treatment efficiency [6,7,8].

The European Federation of Societies for Ultrasound in Medicine and Biology (EFSUMB) guidelines indicate that CEUS is most effective when utilized alongside other traditional sonographic techniques like B-mode and elastography. Regrettably, its integration into routine clinical practice is not yet advised [9].

This paper aims to highlight the role of US elastography, CEUS, and their combined use in the diagnosis and management of malignant thyroid nodules.

## 2. Methods

In the present review, we performed a computerized search using the PubMed database (https://pubmed.ncbi.nlm.nih.gov/, accessed on 31 October 2024), including articles listed up to 31 October 2024. The following keywords were used: contrast-enhanced ultrasound/elastography/thyroid nodules/thyroid malignancy/treatment/lymph nodes. The search was conducted between January 2011 and October 2024, and only English-language articles were included. A total of 829 studies were retrieved, and 85 were included in this article. To eliminate bias, only studies utilizing histological reference as the gold standard were included, and all MESH terms were required to be present in the titles or abstracts.

## 3. Technical Considerations

Assessment of thyroid nodules begins with gray-scale and color Doppler ultrasound that can provide an important amount of information regarding the risk of malignancy due to a combination of suspicious ultrasound characteristics, referred to as the Thyroid Imaging Reporting and Data System (TIRADS). However, the conventional ultrasound has certain limitations, including low reproducibility and dependence on the operator, which can reduce diagnostic value [5,10]. An accurate diagnosis of malignant and benign nodules is essential for prognostic evaluation and to prevent overdiagnosis, overtreatment, and excessive utilization of medical resources [11].

### 3.1. Elastography

Ultrasound elastography techniques are categorized according to the external force employed, the measured quantity, and the manner how results are displayed in compression imaging methods, which utilize internal or external deformation stimuli, and shear wave imaging methods, which employ ultrasound-generated shear wave stimuli [12,13]. Strain elastography (SRE) is considered to have a limited role in thyroid assessment, being influenced by adjacent thyroid tissue and the presence of intralesional calcification. Real-time 2D Shear Wave Elastography (2D-SWE) is considered to be less reliant on user variability compared to qualitative or semi-quantitative quasi-static methods, as it employs acoustic impulses produced by the transducer to assess elasticity rather than external pressure exerted by the operator. For SWE, the stiffness of tissue is quantified as an elasticity index (EI) expressed in kilopascal (kPa) or meter per second (m/s), along with a qualitative color-coded elasticity map [13]. According to the World Federation for Ultrasound in Medicine and Biology (WFUMB), thyroid elastography should be performed using a high-resolution scanner equipped with a 9–15 MHz linear probe [14].

### 3.2. CEUS

CEUS is a minimally invasive US modality approved only in clinical research at the moment that allows the real-time evaluation of the microvascularization of thyroid nodules via microbubbles. CEUS functions both as a diagnostic instrument and a procedural aid in instances of nodular thyroid pathology, facilitating guidance during ablation procedures for thyroid nodules and metastatic lymph nodes [11,15,16]. Two types of contrast agents can be used to assess thyroid pathology: SonoVue^®^ (Bracco Imaging SpA, Milano, Italy) and Sonazoid^®^ (GE Healthcare, Spangereid, Norway). The careful choice of contrast agent is essential for enhancing imaging and refining diagnosis.

SonoVue^®^ is the most prevalent contrast agent available and consists of sulfur hexafluoride microbubbles surrounded by phospholipids and palmitic acid with a diameter between 2 and 10 μm; it is administered intravenously as a small bolus following preparation for CEUS. In comparison with the contrast agents used in computed tomography (CT) or magnetic resonance imaging (MRI), the primary benefit of the CEUS method is its reduced occurrence of adverse effects [4,17].

The Sonazoid^®^ shell consists of hydrogenated egg phosphatidylserine (HEPS) encasing a perfluorobutane (PFB) core. Sonazoid^®^, in comparison with SonoVue^®^, exhibits an additional Kupffer phase or post-vascular phase (defined as the interval of 10 to 30 min following injection) that permits evaluation of focal liver lesions. This phase also allows the evaluation of cervical lymph nodes in patients with thyroid cancer due to the presence of macrophages in the cortex and medulla of the normal lymph nodes [18,19].

Currently, there is no established protocol for the CEUS assessment of thyroid nodules. However, most of the studies suggest the use of a high-frequency linear probe, the setting of the mechanical index < 0.10 to minimize microbubble destruction, and a single focus always placed at the lower portion of the field of view, under the target nodule in order to avoid microbubble disruption. The entire nodule and adjacent normal thyroid tissue should be assessed during the exam. After a B-mode ultrasound is performed to identify the focal lesion of interest, a second-generation contrast agent is administered intravenously as a bolus of 1.0–2.0 mL, followed by a flush of 5–10 mL sodium chloride solution; specific saline and contrast agent dosages differ amongst operators and institutions. To prevent artifacts, the patient must remain motionless, breathe calmly, and not swallow while images are captured for at least 180 s after injection [2,4,11,20].

Qualitative parameters, such as intensity, homogeneity, contrast media uptake, and washout rate, describe nodule vascularization in relation to the surrounding tissue. Following the acquisition of raw data, specialized software can be used to perform more thorough post-processing of the nodule, which includes quantitative analysis; for that, the examiner must place a region of interest (ROI) within the nodule and surrounding parenchyma in order to generate time-intensity curves (TIC), based on which several parameters are evaluated: area of ROI, wash-in slope, time to peak (TTP), peak intensity, area under the curve, mean transit time and washout [2,9]. No singular CEUS feature demonstrates sufficient sensitivity and specificity for malignancy diagnosis [9,16].

## 4. Elastography and CEUS in Characterization of Malignant Thyroid Nodules

In recent decades, the prevalence of thyroid cancer has risen rapidly worldwide, with papillary thyroid carcinoma (PTC) being the most common histological type [21]. The primary objective of the US evaluation of thyroid nodules is to rule out the potential for thyroid cancer, despite the fact that only 7% to 15% of thyroid nodules are malignant [22]. As the conventional US does not yet offer sufficient accuracy for predicting thyroid malignancy, FNA continues to serve as a definitive, minimally invasive diagnostic technique for stratifying the risk of thyroid malignancy with reasonable accuracy. The diagnosis of FNA relies on the Bethesda System for Reporting Thyroid Cytopathology (TBSRTC). Despite the advantages of FNA, there are notable limitations, such as a high incidence of non-diagnostic results (TIR 1 category) related to size and cell sampling or inadequate results and ambiguous cytological interpretations (TIR 3), that make subsequent clinical management challenging [3,23]. There has been significant interest in developing suitable diagnostic algorithms to assess malignancy risk in order to reduce the number of unnecessary biopsies.

International guidelines issued by WFUMB and EFSUMB recommend the ultrasound elastography method as an ancillary imaging modality to the conventional US for differentiating thyroid lesions and monitoring lesions previously identified as benign using FNA [14,24].

Elastography has drawn special interest because several studies have linked the reduced elasticity of the lesions with an increased incidence of malignancy [25,26]. The assessment of thyroid nodule stiffness as a marker for malignancy is said to yield greater diagnostic accuracy relative to various TIRADS. Quantitative methods like SWE, which determine absolute tissue stiffness measurements instead of relative values or ratios, are claimed to be more objective and less reliant on human variability compared to strain elastography [27].

A retrospective study by Azizi et al. evaluated 707 thyroid nodules using Virtual Touch Imaging quantification (VTIQ) and detected a single cut-off value of 3.54 m/s as the maximum shear wave velocity (SWV) for predicting thyroid malignancy with a sensitivity of 79.27% and a specificity of 71.52% [28]. Liu et al. found that the optimal SWE cut-off value of 51.95 kPa for Emax demonstrated a sensitivity of 81.44% and a specificity of 83.19% in differentiating malignant nodules benign nodules [29]. A 2020 meta-analysis indicated that the cut-off values for SWVmax and Emax ranged between 3.51 and 3.52 m/s and 26.6 kPa, respectively; SWVmean ranged from 2.6 to 3.15 m/s; and Emean ranged from 27.65 to 85 kPa across various investigations involving the three manufacturer sub-groups: Toshiba-SWE (T-SWE), VTIQ, and SuperSonic-SWE (S-SWE); the sensitivity and specificity for the previously mentioned manufacturers were the following: 77% and 76% for T-SWE, 72% and 81% for VTIQ, 63% and 81% for S-SWE, respectively, [27]. The complexity of varied SWE index parameters and ideal cut-off thresholds is well documented in the literature. Diverse SWE measurement methodologies may account for the variability.

Chambara et al. demonstrated that the integration of EU-TIRADS with SWE utilizing the standard deviation (SD) parameter preserved a high sensitivity (72.2% vs. 88.9%, *p* > 0.05) and significantly enhanced the specificity (76.5% vs. 55.9%, *p* < 0.01) of EU-TIRADS alone for nodules measuring 1–2 cm; similar results were obtained for nodules exceeding 2 cm [30]. A combination of C-TIRADS and SWE significantly improved the diagnostic efficacy in detecting malignant nodules among category 4a and 4b thyroid nodules with a sensitivity of 83.3%, a specificity of 84%, and an area under the curve (AUC) of 0.870 [31].

A qualitative color pattern SWE developed by Yi et al. showed superior diagnostic efficacy compared to quantitative SWE parameters in distinguishing between benign and malignant ACR-TIRADS 4 or 5 category thyroid nodules measuring ≤ 10 mm. When integrating the qualitative color pattern SWE with ACR-TIRADS scores, utilizing an optimum cut-off value of total points ≥ 8, the thyroid nodules were classified as malignant. The sensitivity, specificity, accuracy, and AUC were 89.90%, 56.94%, 81.11%, and 0.820, respectively. The combination of qualitative color pattern SWE and ACR-TIRADS demonstrated superior diagnostic performance compared to the use of qualitative color pattern SWE alone, with a statistically significant difference (*p* < 0.05) [32]. Therefore, SWE can supplement the B mode findings and potentially improve differentiation between malignant and benign nodules and the selection of patients for FNA [28].

Certain studies indicate that SRE may also serve as an additional ultrasound modality that enhances the diagnostic utility of ultrasounds in evaluating thyroid nodules and potentially decreases the necessity for diagnostic surgeries related to thyroid nodules [33]. Cantisani et al. established a cut-off value of ≥2.0 for strain ratio elastography, indicating a higher likelihood of malignancy [34]. A slightly higher cut-off value of 2.69 for strain ratio nodule/tissue was proposed by Mena et al., yielding a sensitivity of 84%, but only a low specificity of 57% [35]. A prospective study examined 271 thyroid nodules utilizing TIRADS, SRE, and 2D-SWE measured in kPa, categorizing the lesions into three groups based on size: <1 cm, 1–2 cm, and >2 cm. The ideal cut-off value for SRE was 1.92, demonstrating superior performance with a sensitivity of 78.9% for nodules < 1 cm, 87% for nodules 1–2 cm, and 77.8% for nodules > 2 cm; however, the specificity was substantial only for nodules measuring 1–2 cm. This may be attributed to the challenges in targeting smaller nodules and the structural heterogeneity of larger nodules. Combining SRE with TIRADS yielded higher sensitivity (90.4%) compared to TIRADS or SRE alone (59.6% and 82.7%, respectively), albeit with reduced specificity compared to SRE alone [36].

Kyriakidou et al. evaluated the diagnostic accuracy of three distinct elastography techniques: SRE, point-SWE utilizing Acoustic Radiation Force Impulse (ARFI) Imaging, and 2D-SWE for distinguishing thyroid nodules. The research exhibited similar outcomes for ARFI and 2D-SWE in distinguishing thyroid nodules. SE had limited sensitivity and specificity, and the results were not comparable to ARFI [37].

A recent meta-analysis by Cantisani et al. included 72 studies and showed a pooled sensitivity, specificity, and AUC of 84%, 81%, and 0.89, respectively, for qualitative elastography; 83%, 80%, and 0.93 for semi-quantitative elastography and 78%, 81%, and 0.88. for quantitative elastography, confirming that elastography is an effective imaging modality for characterizing thyroid nodules [38]. Table 1 presents the main characteristics of the included studies for ultrasound elastography.

Thyroid nodules are distinguished at CEUS by evaluating qualitative characteristics and measuring quantitative parameters [9]. High-risk features of malignancy on CEUS consist of low enhancement, heterogenous enhancement, ill-defined enhancement border, slow wash-in, and fast wash-out rate [9,17,39]. The low-enhancement pattern is considered the most accurate predictor of malignancy on CEUS, proving excellent sensitivity, specificity, and accuracy rates of 82%, 85%, and 84%, respectively. The primary cause of thyroid malignant tumors exhibiting insufficient blood supply is the intricate neovascularization within the tumors; necrosis and embolus development occur when growth exceeds neovascularization, resulting in low enhancement on CEUS [9,17].

Additional features indicative of malignancy, such as non-homogenous wash-in and wash-out, were observed nearly twice as frequently in carcinomas in a study conducted by Brandenstein et al. TIC perfusion analysis reported that malignant nodules have a significantly lower time to peak (TTP) in comparison with benign nodules both centrally and marginally, indicating a faster and more pronounced uptake of contrast agents in cancers (average TTP values were 13.9 s along malignant nodules margins and 13.2 in their centers, respectively, 19.9 s marginally and 19.7 s centrally for benign nodules; *p* = 0.004 marginally and *p* = 0.003 centrally) [6].

Several studies have reported that nodule size impacts the CEUS assessment and interpretation, with nodules under 10 mm diameter showing absent or no significant contrast media uptake. Small tumors are generally equipped with poor, incomplete neovascularization, which is responsible for insufficient blood supply, resulting in no apparent contrast media uptake. Another possible explanation is the high interstitial fibrosis that is present, especially in papillary thyroid carcinoma [40]. A study by Li et al. evaluated the diagnostic performance of qualitative CEUS parameters for small, solid thyroid nodules ≤ 10 mm and concluded that malignant nodules predominantly exhibited low-enhancement (73.39%), centripetal enhancement (85.18%), indistinct margins (72.06%), irregular shape (81.97%), and heterogenous enhancement (76%). The specificity of CEUS qualitative analysis (89.29%) was markedly superior when compared to the ACR TI-RADS (55.95%) [41].

**Figure 1:**
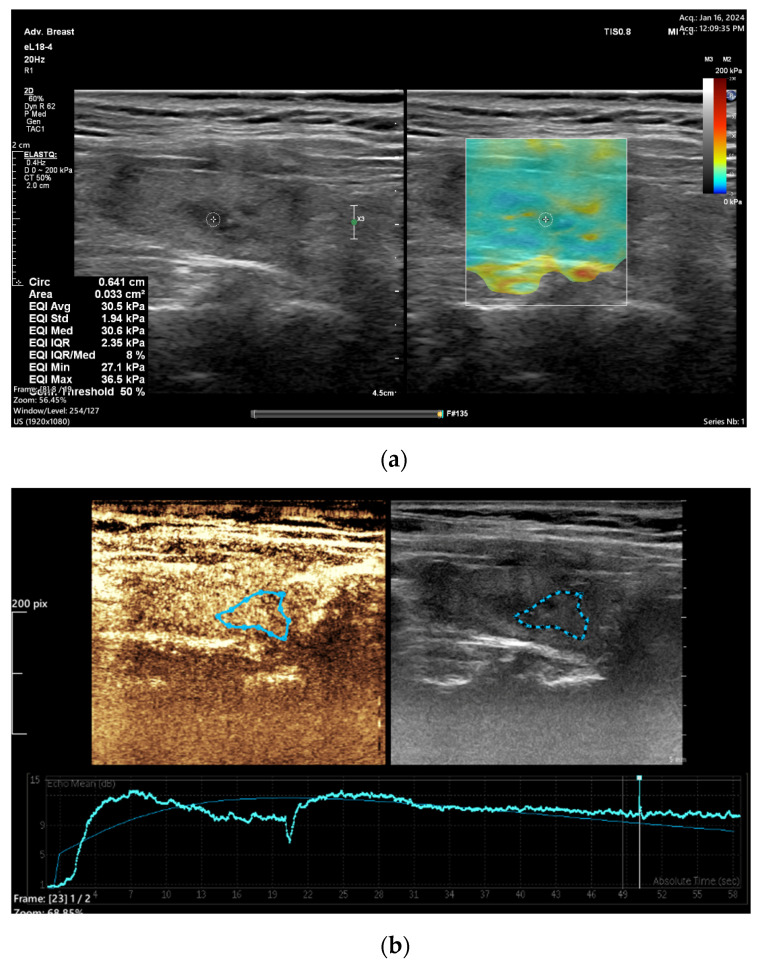
A 66 year old female with papillary thyroid carcinoma overlapped with Hashimoto thyroiditis. (**a**) Quantitative shear wave elastography (SWE) revealed 30.5 kPa; (**b**) qualitative contrast-enhanced ultrasound (CEUS) of the nodule revealed homogenous hyper-enhancement and quantitative CEUS revealed a time to peak of 20.3 s and a peak intensity of 11.58 dB.

**Figure 2:**
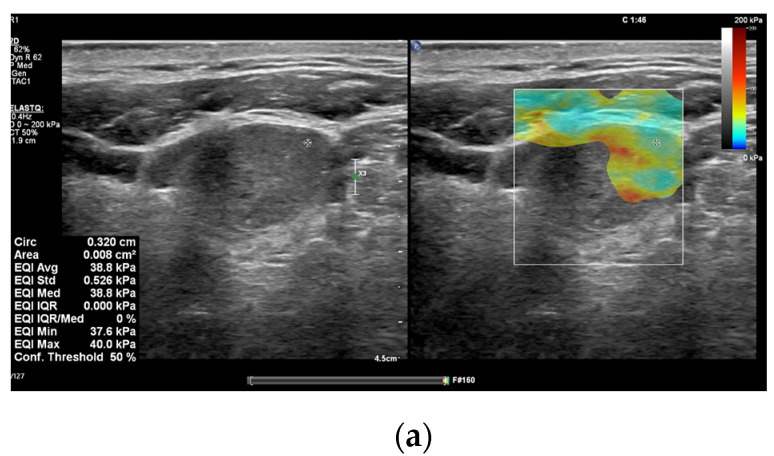

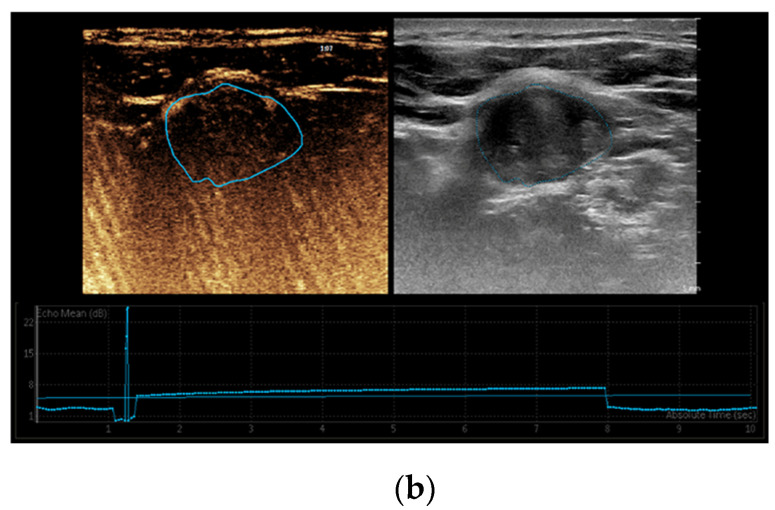
A 52 years old male with multifocal papillary thyroid carcinoma developed on multinodular goiter. (**a**) Quantitative shear wave elastography (SWE) revealed 38.8 kPa; (**b**) qualitative contrast-enhanced ultrasound (CEUS) of the nodule revealed heterogenous hypo-enhancement and quantitative CEUS revealed a time to peak of 10.07 s and a peak intensity of 3.18 dB.

In recent years, many research papers have demonstrated that multiparametric evaluation of focal thyroid lesions using conventional US in association with elastography and CEUS enhances the sensitivity and specificity of ultrasound in predicting thyroid malignancy (Figure 1 and Figure 2).

In a study published by Wang et al., no significant difference (*p* < 0.3282) was observed between ACR-TIRADS and CEUS in differentiating malignant non-hypovascular thyroid nodules. However, the predictive model combining both methods demonstrated superior performance compared to either method alone (*p* < 0.05), achieving a sensitivity of 87%, specificity of 86.2%, and accuracy of 86.6% in the deviation cohort [42]. A prospective multicentric study validated the utility of modified TI-RADS using CEUS for predicting malignant thyroid nodules, obtaining a sensitivity of 93.6% and a specificity of 88.5%. The AUC in this study for the modified TI-RADS was 0.936, and the highest inter-observer agreement was noticed for the taller-than-wide shape and microcalcification (0.94, respectively, 0.93) [43].

A study conducted by Zylka et al. confirmed that using combinations of B-mode features and quantitative contrast patterns in the CEUS examination substantially increases diagnostic precision. Using multivariate logistic regression, the study showed that a combination of features, such as hypoechogenicity (mild or marked) and heterogenous enhancement pattern, was correlated with a threefold increased risks of cancer (OR = 3.36, *p* = 0.004). A similar association was identified for non-smooth margins and hypoenhancement or enhancement equivalent to thyroid parenchyma with a fourfold increase in cancer risk (OR = 4.03, *p* = 0.002) [4].

Ruan et al. conducted a study that included 756 patients and analyzed qualitative US features of the thyroid nodules with univariable and multivariable logistic regression to elaborate CEUS-TIRADS. This new system is based on the ACR-TIRADS, with the primary distinction being the incorporation of CEUS characteristics, such as enhancement direction, peak intensity, ring enhancement, and nodule composition during CEUS examination. The CEUS-TIRADS demonstrated a higher area under the receiver operating characteristic curve of 0.93 (*p* < 0.001), a biopsy yield of malignancy of 66%, and an unnecessary biopsy rate of 34%. This method necessitates prospective multicenter validation prior to clinical implementation [22]. Table 2 presents the main characteristics of the included studies for CEUS.

A recent meta-analysis of 31 articles demonstrated improved diagnostic outcomes when combining CEUS with elastography to differentiate benign and malignant thyroid nodules, with a sensitivity of 0.93 and a specificity of 0.91 [44].

## 5. Elastography and CEUS in Characterization of Indeterminate Thyroid Nodules

The malignancy incidence of thyroid nodules classified as indeterminate is reported to range from 20% to 50%, leading to 50% to 80% of patients undergoing unnecessary diagnostic surgery [45].

Regardless of the ongoing refinement of cytological classifications to enhance the risk categorization of indeterminate thyroid lesions, a considerable proportion of nodules remain without a definitive cytological classification, with some studies showing rates between 2% and 30% [23,46]. Consequently, a substantial percentage of patients undergo unwarranted thyroid surgery, primarily for diagnostic rather than therapeutic reasons, increasing the risk of serious surgical complications.

A second FNA can exclude malignancy in 30–60% of instances, potentially decreasing the number of surgeries [46]. Core needle biopsy (CNB) could be a useful supplemental diagnostic instrument for patients with indeterminate FNA results, providing larger tissue samples and facilitating immunohistochemistry when necessary [46]. However, these are invasive diagnostic methods associated with specific risks. Molecular testing can be used to aid diagnostic accuracy, but it is often limited due to high costs and the invasive nature of FNA to procure tissue samples [47].

Therefore, there is an increasing demand for supplementary diagnostic instruments to more precisely assess the malignancy risk of indeterminate nodules, thereby decreasing the incidence of procedures for non-malignant conditions.

SWE was found to be a valuable diagnostic tool for estimating preoperative malignancy risk in thyroid nodules with indeterminate cytology, showing a sensitivity of 83.9%, a specificity of 79.2%, and a negative predictive value of 95%, with a cut-off SWE value of ≥3.59 m/s [47]. Yoo et al. demonstrated that variations in degrees and patterns of tissue fibrosis among the thyroid nodules appeared to induce distinct patterns of elasticity in SWE, facilitating differentiation between follicular neoplasm (FN) and nodular hyperplasia (NH) in follicular pattern lesions of thyroid nodules that represent over 50% of FNA samples. FN exhibited a reduced maximum elasticity value (measured in kPa) compared to the NH group (*p* < 0.05) [45], due to a lower level of fibrosis. Furthermore, Zhang et al. reported that combining SWE and ACR-TIRADS improved diagnostic sensitivity and accuracy, aiding in differentiating benign and malignant thyroid nodules with indeterminate FNA cytology [48].

Celleti et al. proclaimed the superiority of SRE assessment over SWE in risk stratification of indeterminate nodules, with a sensitivity of 85.7% vs. 57.1%, a specificity of 94.1% vs. 79.4, and an AUC of 0.899 vs. 0.683, respectively. The combination of SRE and K-TIRADS demonstrated improved NPV relative to K-TIRADS alone (96.3% vs. 87.5%), particularly for nodules measuring ≤ 1 cm, with both specificity and NPV reaching 100% [49].

The efficacy of different elastography techniques in distinguishing benign from malignant indeterminate thyroid nodules was assessed in a meta-analysis conducted by Qui et al., reporting an overall elastography sensitivity of 76.6% and a specificity of 86.7%. In the subgroup analysis, sensitivity and specificity were 83.8%, 87.2% for SWE, 81.3%, 89.4% for SR, and 71.5% and 85.3% for real-time elastography [50]. A recent meta-analysis from 2023 conducted by the same group of authors encompassing seven studies evaluated the role of SWE, showing a sensitivity of 76.6% and a specificity of 86,7% in distinguishing indeterminate nodules; the small number of articles (only three regarding SWE) and the sample size in the previous meta-analysis may explain the differences [51].

In a multiparametric assessment of indeterminate thyroid nodules, Gay et al. found that SE values had a better correlation with histological outcome (*p* = 0.05) compared to SWE values (*p* = 0.20). CEUS quantitative parameters, TTP, and peak index (PI), proved to be insufficient in differentiating benign from malignant lesions (*p* = 0.97 and *p* = 0.72, respectively) [46].

So far, only a few papers have investigated the reliability of CEUS as a predictor tool of malignancy/benignancy in patients with thyroid indeterminate nodules. A recent study conducted by Zhang et al. found that combining CEUS with Chinese TI-RADS improves AUC, diagnostic sensitivity, and accuracy and facilitates the differentiation of indeterminate FNA cytological nodules (all *p* < 0.05) [52]. CEUS characterization of indeterminate thyroid nodules could improve the diagnostic process of indeterminate thyroid nodules and may reduce the number of FNA; nonetheless, substantial prospective studies are required to validate these findings.

## 6. Preoperative Prognostic Features Evaluation Using Elastography and CEUS

According to the American Thyroid Association (ATA) guidelines, all patients suspected of having thyroid neoplasm should undergo a preoperative neck ultrasound to evaluate the primary tumor, determine lymph node involvement, and provide information to guide surgery extent. Precise staging is crucial for assessing prognosis and customizing treatment for patients with differentiated thyroid cancer (DTC) [21].

Thyroid extracapsular extension (ECE) and the existence of metastatic lymph nodes are recognized to elevate the risk of locoregional recurrence in papillary thyroid carcinoma [53].

ECE is regarded as an unfavorable prognostic factor in thyroid carcinoma that plays a crucial role in assessing tumor staging and surgical management of patients with PTC [21,54]. The diagnostic accuracy of conventional US in the detection of ECE has been evaluated in several previous studies. A good sensitivity of 86.4%, with a low specificity of only 29.8%, and a diagnostic odds ratio (DOR) of 2.68 was observed in a study conducted by Ramundo et al. using gray-scale and color Doppler imaging that employed a “nonrestrictive definition” (i.e., the nodule abuts the thyroid capsule with or without signs of disruption) to evaluate ECE. For the “very restrictive definition” (i.e., the nodule disrupts the capsule and invades soft tissue and/or perithyroidal muscles), the study reported a specificity of 100%; however, it exhibited exceedingly low sensitivity (6,8%), and its overall diagnostic efficacy was not significant (DOR of 14.25) [55]. A meta-analysis from 2023 had reached a sensitivity of 86.4%, a specificity of 51.2%, and a DOR of 5.31 [56].

Based on earlier studies, CEUS could help provide more precise ECE detection than conventional US, benefiting surgical treatment of patients with PTC [57,58]. In a study by Zhang et al., 124 PTC nodules were analyzed with CEUS and showed that CEUS outperforms conventional US in identifying ECE in PTC (AUC: 79.4% vs. 65.8%; *p* = 0.02). The study also evaluated the correlation between ECE and nodule enhancement patterns in patients with PTC and concluded that hyper-enhanced nodules exhibited the highest prevalence of single capsule invasion (41.9%), whereas hypo-enhanced nodules demonstrated a greater incidence of ECE (47.4%) [59].

PTC is distinguished by a predisposition to cervical lymph node metastasis (CLNM) with an incidence that can reach approximately 20–50% [60]. Early detection of CLNM is essential in managing DTC, as it affects surgical indications and extent, thereby influencing recurrence risk and overall survival [61]. ATA recommendations indicate that conventional US is considered the preferred method for assessing CLNM over CT and MRI due to its reduced costs and efficiency. The central B-mode US features suggestive of abnormal metastatic lymph nodes comprise enlargement, hyperechogenicity, absence of the fatty hilum, a round shape, cystic areas, calcifications, and peripheral flow on the Doppler mode US [21].

However, conventional ultrasound diagnostic efficacy is limited in differentiating between reactive and metastatic lymph nodes and identifying specific anatomically concealed lymph nodes or small lymph nodes presenting cystic degeneration and focal hyperechogenicity. The value of routine US is controversial because of its inconsistent sensitivity, which fluctuates from 33% to 70% [62].

Due to the frequent infiltration of tumor cells, tumoral necrosis, calcification in malignant lymph nodes, and absence in benign lymph nodes, elastography can yield critical clinical insights for distinguishing between lymph node conditions [63]. Jung et al. demonstrated that SWE-EI of Emean, Emin, Emax, and Emean-m were considerably elevated in metastatic lymph nodes compared to benign lymph nodes in patients with PTC. The highest specificity (100%) and PPV (100%) were obtained with Emin (cut-off value of 24.0 kPa), the highest sensitivity (84.3%) with Emax (cut-off value of 57.0 kPa), and the highest accuracy (72.6%) with Emean (cut-off value of 29.0 kPa) [64]. Metastatic lymph nodes with extranodal invasion exhibited significantly elevated SWE-EI values compared to intranodal metastatic lesions [64]. These results are supported by another study conducted by Li et al. showing that SWE can predict CLNM in people with PTC; multivariate analysis showed that Emax > 59 kPa, along with microcalcification, extrathyroidal extension, and multifocality were independent risk factors for predicting CLNM [65]. Nevertheless, Han et al. reported that combining SWE with B-mode US offers no benefits in terms of prediction of ECE and CLNM in patients with PTC relative to B-mode US alone [53].

CEUS assesses the microvascular flow of lymph nodes in real-time and could refine ultrasound diagnostic accuracy for CLNM in patients with PTC [66,67]. Inflammatory or reactive lymph nodes often exhibit intense homogenous enhancement, whereas perfusion defects indicate metastatic involvement; minimal or absent perfusion may be evident in extensive metastatic infiltration, indicating the existence of significant necrotic regions. Also, CEUS can effectively characterize focal cortical thickening depicted on the gray-scale US in patients with suspected nodal metastatic involvement. Metastatic deposits present reduced vascularization compared to the neighboring nodal parenchyma, a characteristic that is more pronounced during the parenchymal phase due to early contrast wash-out. Conversely, localized thickening in benign nodes frequently manifests, enhancing characteristics similar to those of the surrounding nodal tissue. Differences regarding perfusion kinetics have been studied, and it has been shown that benign nodes present centrifugal progression of enhancement compared with metastatic nodes that tend to centripetal enhancement [66].

The preoperative diagnostic accuracy of CEUS in differentiating between benign and metastatic cervical lymph nodes in patients with PTC was analyzed in several studies. Hong et al. pointed out that metastatic lymph nodes more frequently manifested centripetal or asynchronous perfusion, hyperenhancement heterogeneous enhancement, perfusion defects, and ring-enhancing margins in comparison with benign nodes; for conventional US sensitivity and specificity, 86.4% and 73.9%; for CEUS, 84.6% and 94.3%; and conventional US combined with CEUS, 92.6% and 91.7%, respectively [68].

Luo et al. reported a higher sensitivity and diagnostic accuracy of quantitative parameters in comparison to qualitative parameters (75% vs. 55% and 83.6% vs. 76.1%, respectively); PI was significantly higher in the metastatic group compared to the benign group (13.5 dB vs. 11.08 dB; *p* = 0.000); TTP and time from PI to one half (DT/2) were significantly longer in the metastatic group compared to the benign group (65.36 s vs. 52.04 s, 1820.8 dBs vs. 1050 dBs, respectively; *p* = 0.000 for both), showing a slow wash-in and wash-out [69]. A meta-analysis by Li et al. showed that the pooled sensitivity, specificity, and AUC of CEUS were 80%, 90%, and 0.90, respectively, confirming its high efficacy in diagnosing CLNM of PTC [67].

Lymphatic contrast-enhanced ultrasound (LCEUS) has gained attention in the last few years, being applied in detecting and differentiating sentinel lymph nodes in patients with breast cancer [70]. Wei et al. investigated a combination of LCEUS and intravenous CEUS (IVCEUS) using Sonazoid^®^ for the detection of CLNM originating from PTC; for the LCEUS, the contrast agent was injected into the superficial thyroid parenchyma in front of the tumor allowing assessment of lymph nodes and lymphatic vessels. Malignant nodes displayed interruption of the bright ring and perfusion defects, while benign nodules presented the complete bright ring and homogenous perfusion on the LCEUS; perfusion defect was associated with the metastatic foci in the medulla, and the disruption of the bright ring was linked to tumor seeding in the marginal sinus (all *p* values < 0.05). The combination of LCEUS and IVCEUS markedly improved the diagnostic efficacy compared to either method alone [71]. A similar study confirmed that perfusion defects and interruption of the bright ring are LCEUS features highly suggestive of malignancy [72].

In addition, Xiao et al. demonstrated that the post-vascular phase of CEUS using Sonazoid^®^ helps diagnose suspicious small lateral cervical lymph nodes (short-axis diameter ≤ 8 mm) in patients with PTC. Perfusion defect showed a specificity of 96% in the vascular phase, and the NPV of the non-isoenhancement (i.e., hypoenhancement, partial enhancement, and no enhancement) reached 100% in the postvascular phase [19].

Nevertheless, a recent study conducted by Yang et al., which included 965 patients (527 classified as clinically node-negative and 438 as clinically node-positive prior to surgery), revealed that the accuracy of contrast-enhanced ultrasound (CEUS) in evaluating cervical lymph node metastasis in patients with PTC was merely 62.8%, markedly lower than that reported in earlier studies. A potential cause is that previous studies were analyzed in a reasonably limited cohort. In light of these findings, it is advisable to consider other methods, such as contrast-enhanced CT/MR and an intraoperative pathological biopsy of questionable lymph nodes, alongside CEUS [73].

## 7. Treatment Evaluation of Thyroid Malignancy and CLNM Using Elastography and CEUS

Despite significant achievements in surgical techniques for DTC throughout recent decades, ranging from conventional open thyroidectomy to endoscopic thyroidectomy, postoperative complications such as permanent recurrent laryngeal nerve palsy, hypoparathyroidism, and infection continue to be reported in contemporary research. Over the last years, active surveillance (AS) has been proposed as a viable alternative for surgical excision in patients with PTC with diameter ≤ 10 mm, defined as intrathyroidal papillary microcarcinomas (PTMC), particularly individuals who are at high surgical risk and/or anticipated limited longevity (Figure 2). Regardless of the encouraging results of AS, implementation in clinical practice is significantly impacted by psychosocial factors, patient comorbidities, and motivation [21,74]. Therefore, some patients prefer to tolerate relatively aggressive interventions as an alternative to AS.

Thermal ablation procedures are seen as a valuable supplement to the existing therapeutic modalities that might increase the patient’s experience and compliance by reducing the invasiveness of treatment in patients with benign thyroid nodules, recurrent thyroid malignancy, and CLNM [5,75]. The most utilized image-guided thermal ablation techniques available for solid- and mixed-structure thyroid nodules are next: radiofrequency ablation (RFA), microwave ablation (MWA), laser ablation (LA), and high-intensity focused ultrasound (HIFU). Thermal ablation aims to achieve irreversible coagulative necrosis of the thyroid nodule, resulting in a subsequent decrease in volume. At ablation temperatures of 60 °C, vessels with a diameter of less than 3 mm are obliterated by heat, hence, ceasing the supply to the nodules. This effect is missing in vessels above 4 mm in diameter, where a loss of energy, also called a “heat sink”, is observed [76].

MWA is a minimally invasive procedure extensively utilized in clinical practice for benign lesions. It facilitates a substantial reduction in operating time, recovery duration, and hospital stay and provides adequate therapeutic outcomes [75]. The role of MWA has also been studied in the treatment of low-risk PTMC (clinical stage of T1N0M0) and proved to be a safe and long-term effective method [77,78,79].

In a prospective study by Yue et al., 119 unifocal PTMC patients underwent MWA under US guidance. A significant tumor volume reduction was registered from the immediate post-MWA (1.84 ± 1.03 mL) to the final assessment (0.01 ± 0.04 mL), yielding a mean volume of reduction ratio of 99.4 ± 2.2%, with 107 patients (93.9%) achieving complete remission. The follow-up period subsequent to MWA was 37.2 ± 20.9 months [77]. Hu et al. found that age, calcification type, and initial maximum diameter were independent prognostic factors predicting ablation zone status after MWA [79].

A study by Zhou et al. showed that MWA and LA are safe and efficient methods for treating patients with unifocal PTMC. The mean volume decreased from 51.9 ± 40.8 to 0.2 ± 1.0 mm^3^ in the MWA group and from 38.5 ± 43.0 to 1.3 ± 3.8 mm^3^ in the LA group (*p* < 0.05 for both). The complication rates were comparable between the MWA group (9.1%) and the LA group (2.9%) (*p* > 0.05) [80].

Li et al. compared the outcomes for patients with PTMC who underwent MWA versus surgery and found no statistically significant differences between the two groups in terms of PTM recurrence, lymph node metastasis, and disease-free survival; the incidence of transitory hypoparathyroidism was markedly reduced in the MWA group compared to the surgery group (*p* < 0.001) [81].

Compared with other thermal ablation procedures, MWA advantages include reduced ablation times, a lower heat-sink effect of nearby vessels, and total eradication of tumor cells [82].

PTMC is an indolent, slowly progressing disease that necessitates an extended and more rigorous follow-up period to assess treatment success [78]. Vascularity and serum thyroglobulin (Tg) concentrations are the main parameters for evaluating the ablated tissue. Careful monitoring of the thermal ablation area by different imaging modalities (computed tomography, magnetic resonance, color Doppler US, or CEUS) is essential to ensure the tumor is entirely ablated promptly after treatment and to identify early recurrences that may require treatment [83].

CEUS can be used to accurately delineate between viable and nonviable tissue of the ablated region in thyroid nodules subjected to thermal ablation procedures. Furthermore, US contrast agents can be directly delivered to facilitate a routine US examination [79], [80]. A favorable outcome of the ablation procedure based on the imaging findings is considered when: (1) no contrast enhancement was noted around or within the lesion; (2) the border of the ablation zone was distinct and smooth; and (3) the ablation area extended beyond the tumor margin. In the event of incomplete ablation, further ablation treatment must be accomplished [80,84].

Zhang et al. assessed the magnitude of the ablation zone following percutaneous LA with CEUS seven days post-PLA. They observed that the dimensions of the resultant necrotic area were more significant than those recorded 10–20 min after PLA. The results suggest that the immediate post-procedural extent of the ablated region may not accurately reflect the ultimate necrotic volume following thermal ablation, necessitating a short-term repeated CEUS. This is likely due to sub-lethally wounded tissue near the periphery of the ablation zone, which is known to ultimately undergo necrosis following thermal ablation [84].

## 8. Conclusions

The combination of elastography and CEUS might improve the sensitivity and diagnostic precision of malignant thyroid nodules compared to the use of either CEUS or elastography alone. It could serve as a non-invasive, reliable, and efficient approach for the diagnosis and treatment of malignant thyroid nodules.

## Figures and Tables

**Table 1:** Main characteristics of the included studies for ultrasound elastography.

Author	Year of Publication	Nodules	Diagnosis	Sensitivity (%)	Specificity (%)	PPV (%)	NPV (%)	AUC
Azizi [28]	2015	707	Quantitative (SWE − VTIQ)	79	72	27	96	-
Liu [29]	2017	313	Quantitative (SWE)	81	83	89	73	0.88
Chambara [30]	2022	126	Quantitative (SWE) + EU-TIRADS	72 (1–2 cm) 71 (>2 cm)	76 (1–2 cm) 96 (>2 cm)	- 83	- 92	0.74 0.84
Li [31]	2023	409	Quantitative (SWE) + C-TIRADS	83	84	85	83	0.87
Yi [32]	2023	270	Qualitative (SWE color pattern score) + ACR-TIRADS	89	57	-	-	0.82
Cantisani [34]	2012	97	Semi-quantitative (SR)	97	92	88	98	-
Cantisani [36]	2019	243	Semi-quantitative (SR) Quantitative (SWE)	83 67	93 83	75 51	95 90	0.87 0.75
Kyriakidou [37]	2018	84	Qualitative (SE) Quantitative (point-SWE -ARFI) Quantitative (2D-SWE)	73 90 73	70 79 67	- - -	- - -	- - -
Cantisani [38] (meta-analysis—72 studies)	2022	14,015	Qualitative Semi-quantitative Quantitative	84 83 78	81 80 81	- - -	- - -	0.89 0.93 0.87

**Table 2:** Main characteristics of the included studies for CEUS.

Author	Year of Publication	Nodules	Diagnosis	Sensitivity (%)	Specificity (%)	PPV (%)	NPV (%)	AUC
Li [41]	2020	185	CEUS	86	89	91	84	0.91
Wang [42]	2021	217	CEUS + ACR-TI-RADS	87	86	86	87	0.92
Zhou [43] (multi-center study)	2023	4532	CEUS + modified TIRADS	94	88	90	94	0.93
Ruan [22]	2022	801	CEUS-TIRADS	89	87	85	90	0.93
Liu [44] (meta-analysis—31 studies)	2024	4718	CEUS + Elastography	93	91	83	85	0.97
